# The functional role of ants in no‐till cropping systems

**DOI:** 10.1002/ps.71152

**Published:** 2026-07-28

**Authors:** Adegboyega O. Fajemisin, Andrew V. Suarez, John F. Tooker

**Affiliations:** ^1^ Department of Entomology The Pennsylvania State University University Park PA USA; ^2^ Department of Entomology, School of Integrative Biology University of Illinois Urbana‐Champaign Urbana IL USA

**Keywords:** no‐till agriculture, formicidae, biological control, ecosystem services, conservation agriculture

## Abstract

Ants dominate terrestrial ecosystems yet are often overlooked in temperate annual cropping systems. These social insects provide valuable ecosystem services in tropical and subtropical agriculture, but their potential roles in temperate row crops have received less attention. This review examines how no‐till farming practices create opportunities for ants to establish and contribute to pest suppression, weed‐seed removal, and soil improvement in temperate row crops. Unlike conventional tillage that may destroy ant colonies, no‐till systems preserve nest architecture and foraging networks, allowing ant populations to persist across growing seasons. Evidence from multiple regions demonstrates that no‐till fields support greater ant abundance, species richness, and predation activity than tilled systems. Cover crops further enhance ant communities by providing habitat complexity and alternative prey, while integrated pest management preserves beneficial populations through judicious insecticide use. Ants offer multiple mechanisms for pest control, including direct predation, non‐consumptive deterrence, and season‐long activity when other natural enemies are scarce. Their soil engineering activities create biopores that improve water infiltration and nutrient transport. Despite these potential benefits, critical knowledge gaps persist regarding ant community composition, seasonal activity patterns, and quantitative impacts on economically important pests in temperate systems. Future research should apply molecular gut content analysis and stable isotope techniques to reveal cryptic trophic interactions, develop economic valuations of ant‐mediated services, and create practical decision‐support tools for farmers. As agriculture faces pressure to reduce environmental impacts while maintaining productivity, conservation practices that support ant communities represent an underutilized strategy for sustainable intensification in temperate cropping systems. © 2026 The Author(s). *Pest Management Science* published by John Wiley & Sons Ltd on behalf of Society of Chemical Industry.

## Graphical abstract

Ants provide valuable ecosystem services in tropical agriculture, but their roles in temperate row crops remain poorly understood. Conservation practices such as no‐till farming and cover cropping support ant communities that contribute to pest suppression, weed seed removal, and soil quality, which can increase crop productivity.

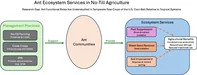

## 1 INTRODUCTION

Ants are ecosystem engineers that shape the structure and function of many terrestrial communities and habitats.^1^, ^2^, ^3^, ^4^ As engineers, ants modify their environment and influence the abundance and distribution of other terrestrial animals.^5^, ^6^ In natural systems, these social insects enrich soil, help cycle nutrients, disperse seeds, and can promote or limit herbivore populations.^7^, ^8^, ^9^, ^10^

Their varied ecological functions, which are well‐documented in natural habitats,^1^ have led to interest and extensive research on how to harness their potential benefits (and avoid costs) for agricultural systems, particularly in tropical and subtropical regions.^11^ Farmers in Asia have used *Oecophylla* weaver ants to control orchard pests for centuries. These ants rank among the earliest documented agents of biological control.^12^, ^13^ In perennial production systems of the tropics and subtropics, such as coffee, citrus, and cocoa plantations, ants can impose effective biological control that reduces herbivory and protects yields.^14^, ^15^, ^16^, ^17^ Similarly, in subtropical annual crops like cotton and rice, certain ant species in the genus *Solenopsis* can provide clear benefits as predators of key pests.^5^, ^11^, ^18^, ^19^ In soybean, the role of ants appears less consistent. In Florida, diverse ant communities provided pest control when fire ants were absent.^20^ In Georgia, soybean plots with fewer ants did not differ in their pest populations.^21^ More broadly, ants can function as important predators while simultaneously farming sap‐feeding insects (Insecta: Hemiptera), which can either harm or benefit crops.^22^, ^23^, ^24^, ^25^ These interactions illustrate the complexities of ant‐mediated effects in agricultural systems. Nevertheless, the beneficial influence of ants in warmer agricultural systems raises an intriguing question: Do ants have potential to play similarly important roles in temperate annual cropping systems? If not, why not, and could cropping systems be modified to be more welcoming to beneficial ant taxa?

Despite well‐documented benefits of ants in tropical and subtropical agriculture (Fig. 1 and Supporting Information, Table S1), their potential roles in annual cropping systems in temperate regions have received little attention.^26^ On the other hand, however, temperate regions likely support lower ant abundance and diversity than tropical and subtropical regions, potentially because colder temperatures likely shorten periods of ant activity and reduce foraging.^27^, ^28^ As a result, ants in temperate regions perhaps exert weaker effects on ecological functions in agricultural systems, though this remains to be tested.

**Figure 1:**
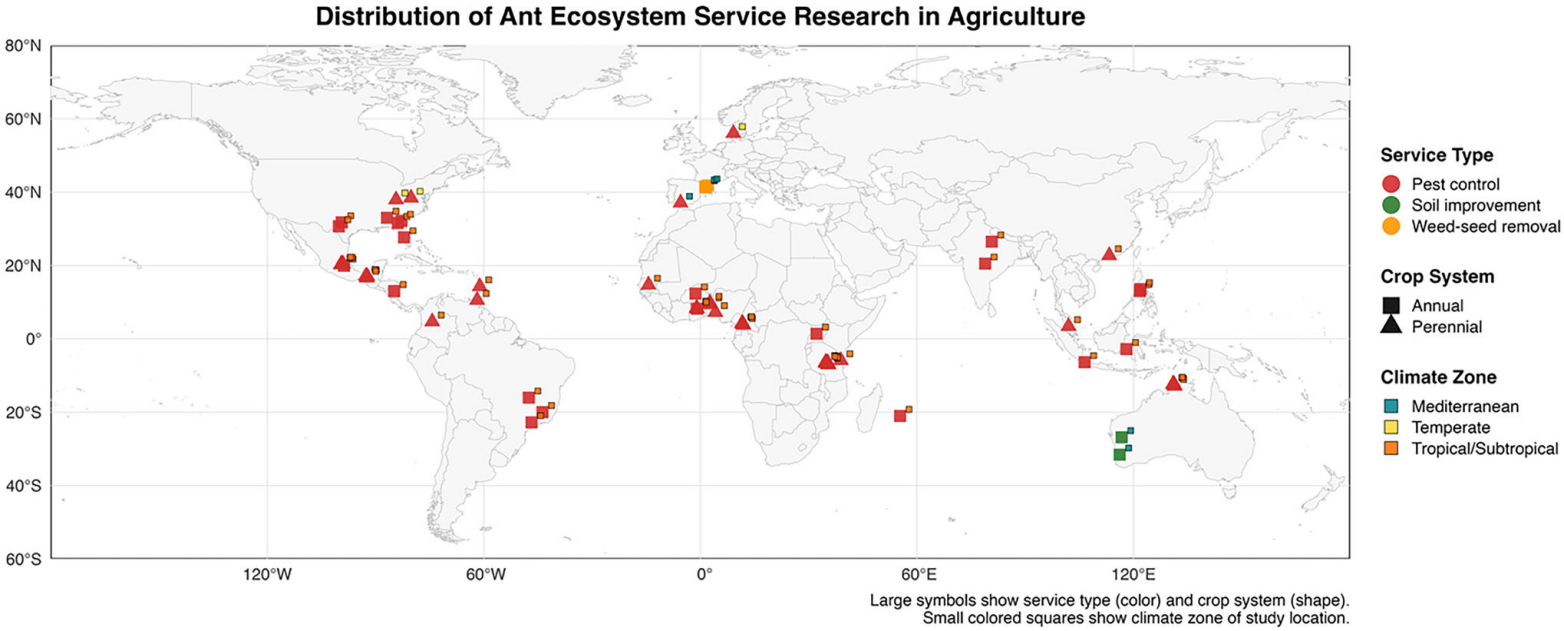
Distribution of examples of research on ecosystem services provided by ants in agriculture. The map shows study locations with large symbols coded by service type (color) and crop system (shape). Small adjacent squares show the climate zone of each location. Supporting Information, Table S1 provides additional details for each of these studies.

Nevertheless, based on their value to other agricultural systems,^11^, ^29^, ^30^ potential benefits of ants warrant investigation in temperate annual cropping systems,^26^ but attention needs to focus on types of farms where they have an opportunity to contribute. For the most part, researchers have ignored ants in large acreage annual crops, such as corn and soybeans of the northern USA, likely because historic and ongoing tillage has reduced ant abundance in fields.^31^, ^32^ In the eastern portion of the US Corn Belt, however, no‐till farming practices have been well adopted^33^ and some research in this region has supported the potential of ants to colonize no‐till crop fields and contribute to ecological functioning of crop fields.^34^ No‐till farms, or farms that have adopted other forms of conservation tillage, hold potential to host substantial numbers of ant colonies because they experience less disturbance from tillage, and ant populations and communities benefit from stable habitats.^31^, ^32^ Moreover, farms that use conservation tillage and have adopted integrated pest management (IPM) may hold even greater potential to host substantial numbers of ant colonies because they should limit their use of unnecessary insecticides,^32^, ^35^, ^36^, ^37^, ^38^ though available studies provide limited support for this hypothesis.

The purpose of this review is to characterize where and how ant communities contribute to ecosystem services in large‐acreage crops of the northern Corn Belt, a region where their role remains largely overlooked despite their potential significance. Our analysis of the literature revealed that studies that have quantified ecosystem services of ants have been concentrated in tropical and subtropical perennial systems, whereas few have examined annual crops in temperate regions (Fig. 1 and Supporting Information, Table S1). While discussing each of these studies is beyond our scope of our review, this imbalance highlights the limited understanding of the roles that ants play in temperate agroecosystems. We aim to identify which services ants likely provide in these systems, drawing parallels from various agricultural systems to understand their potential contribution to pest management and other ecosystem functions in temperate annual crops. By synthesizing current knowledge of ants in agricultural systems, with particular focus on no‐till farms, we seek to understand how researchers, farmers, and other agricultural professionals can maximize ant contributions to create more sustainable and resilient agricultural practices. This investigation will help us understand how management practices can better accommodate and enhance the beneficial roles of ants in northern corn belt agriculture.

## 2 ANTS AND THE ROLES THEY PLAY IN AGRICULTURAL SETTINGS

Ants (Formicidae) rank among the most abundant and diverse animals on Earth, often comprising 50–80% of animal biomass in terrestrial ecosystems.^1^, ^5^, ^27^, ^39^ This ecosystem dominance extends into agricultural landscapes, where ants influence crop production through multiple pathways. While certain species, such as leaf‐cutting ants, can be pests by defoliating plants,^40^ many others provide significant benefits to agricultural systems through their varied ecological roles. Ants function simultaneously as predators, soil engineers, and seed dispersers.^11^, ^41^, ^42^, ^43^ Most ant species are omnivores that consume both animal prey and plant resources.^1^ This broad diet allows ants to occupy diverse trophic roles and influence agricultural systems in both positive and negative ways.^24^, ^44^, ^45^ As predators, they can significantly reduce densities of herbivore populations and associated plant damage.^11^ In a global meta‐analysis on the effects of ants on pest control, ants were found to decrease abundance of non‐honeydew‐producing pests, decreasing plant damage while improving crop yield.^46^ These predatory interactions, however, occur within complex ecological networks. Some ants form mutualistic relationships with honeydew‐producing insects, which can either harm or benefit plants by protecting pests or by sustaining presence of ants for broader pest suppression.^24^ In subtropical regions of the USA, the red imported fire ant (*Solenopsis invicta*) illustrates this duality because it can be a key predator of cotton pests while sometimes promoting aphid outbreaks by protecting aphid populations.^23^, ^24^ In northern regions, including the US Corn Belt, ant‐crop interactions are less studied. While ants occasionally tend aphids in corn and soybean systems, at least in Mid‐Atlantic field crops, these interactions rarely appear to influence crop productivity at the field scale (J.F. Tooker, personal observation).

Better understanding of how ants shape their environment would enhance our knowledge of crop field functioning. Beyond their contributions to pest control and seed removal, ants modify soil structure and composition.^11^, ^41^, ^42^, ^43^ Through nest construction, ants create biopores that facilitate movement of air, water, and nutrients.^47^ In some ecosystems, ants can rival earthworms in the volume of soil they move.^48^ These modifications can reduce soil erosion, enhance nutrient transport, and facilitate root growth.^49^ Additionally, ant activities can significantly affect soil microbial communities, potentially influencing nutrient cycling and soil quality.^50^, ^51^, ^52^ Ant nests also host distinct microbial communities that differ from surrounding soils in composition and activity, which can enhance nutrient turnover and soil fertility.^53^, ^54^

Continuous ant activity also supports season‐long pest suppression. Because colonies remain active throughout the growing season, they can suppress pests when other natural enemies are less abundant.^26^, ^29^ Their foraging and recruitment behavior, facilitated by pheromone trails, enables a rapid collective response to pest outbreaks.^55^, ^56^ In addition to direct consumption, ant activity can also cause non‐consumptive effects; they can disrupt pest feeding and oviposition, and also deter pest colonization through chemical cues.^57^, ^58^, ^59^, ^60^, ^61^ For example, weaver ants (*Oecophylla smaragdina*) release 1‐octanone, which repels and deters oviposition by fruit flies.^62^ These behavioral responses may enhance pest suppression beyond direct predation.

Belowground, ants also prey on pest life stages that develop in soil or crop residue.^63^ Persistent tunnel networks allow them to locate and consume larvae of pests that feed on roots or dwell in soil, such as black cutworms and Japanese beetle grubs.^63^ These belowground interactions extend the influence of ants beyond soil surface, unlike many arthropod predators in agricultural systems. Together, these diverse ecological roles highlight the potential of ants to influence multiple processes in agricultural systems.

### 2.1 No‐till farming and conservation agriculture

Tillage has been a cornerstone of agriculture, serving as a historically essential practice for seedbed preparation and weed control across diverse farming systems worldwide.^64^ Traditional tillage operations provide several advantages: tillage warms soil and allows earlier planting in cooler climates, disrupts weeds and reduces competition, and inverts soil to interrupt the life cycles of some pests, including invertebrates and plant pathogens.^65^, ^66^, ^67^, ^68^, ^69^ Tillage, however, has some downsides because it increases labor and energy costs, and accelerates soil erosion and decomposition of organic matter.^70^ In some regions, these negative effects have prompted widespread adoption of conservation tillage systems, which minimize soil disturbance while maintaining crop productivity.^71^ Modern conservation practices became widespread through technological advancements, including herbicide‐resistant crop varieties and specialized planting equipment for high‐residue systems.^64^ Adoption of conservation tillage varies geographically in the USA, with drought‐prone regions and areas around key surface waters showing the highest rates of adoption. The Mid‐Atlantic region of the USA has particularly high adoption rates, with some areas reaching 75% no‐till or more for large‐acreage crops, providing valuable research opportunities for understanding no‐till systems.^33^, ^72^ No‐till adoption in Mid‐Atlantic states tends to be long‐term no‐till, rather than occasional no‐till, and has been driven by regional and local initiatives to reduce sediment and nutrient run‐off into streams and rivers that flow into the Chesapeake Bay.^73^

Advocates for reduced tillage often emphasize labor and energy‐cost savings along with soil conservation benefits, including reduced erosion and improved water‐holding capacity.^70^ However, an additional, often overlooked, potential benefit involves higher levels of pest management from conserved natural‐enemy communities. Many natural enemies, including predators, parasitoids, and entomopathogens, spend part of their lifecycle belowground. By eliminating soil disturbance, no‐till farming helps maintain these beneficial organisms, which can significantly reduce pest populations.^74^, ^75^, ^76^ A recent meta‐analysis tested whether invertebrate pests and natural enemies differed in abundance between tilled and no‐tilled fields.^77^ Contrary to beliefs of many agricultural professionals that no‐till fields harbor more pests,^78^ this analysis found that foliage‐based pests were more abundant in tilled system, but soil‐based pests were equally abundant in both tilled and no‐till fields. Notably, however, natural enemies, particularly generalist predators, were more abundant in no‐till fields, where habitat stability allows their populations to grow and persist.^77^ Despite this evidence for natural‐enemy conservation in no‐till systems, many classic studies on arthropod communities of no‐till fields appear to have largely ignored ants,^75^, ^79^ suggesting a significant gap in our understanding of these important predators in temperate agricultural systems.

No‐till farming protects ant nests and colonies that tillage would otherwise disturb.^32^, ^35^, ^36^ A study conducted across 90 agricultural sites in North Carolina and Virginia demonstrated significant differences in community composition of ants between tillage systems.^35^ The study found that 13 out of 17 common ant species occurred more frequently in fields using conservation tillage (e.g., no‐till, strip‐till, ridge‐till, and mulch‐till) than in those using conventional tillage.^35^ No‐till appears to provide more stable nesting sites, allowing interactions with other species to structure communities, rather than having communities driven by disturbance from farm management.^35^ Moreover, studies comparing ant communities between tillage systems have found greater species richness in no‐till fields, suggesting these systems can build and then maintain diverse ant assemblages with potentially complementary ecological functions.^35^, ^36^, ^80^ For example, over 4 years in annual crops in Brazil, no‐till systems had higher ant abundance, species richness, and diversity than conventional tillage systems.^36^ While most of these results come from tropical regions, evidence from temperate regions also shows that no‐till practices can substantially increase ant abundance and diversity,^35^ suggesting that no‐till may support ant communities across a broad range of climates.

The long‐term stability of continuous no‐till systems may benefit certain functional groups of ants. Seed‐harvesting ants, which can contribute to weed management, establish more effectively in undisturbed soils where their granaries and foraging tunnels remain intact.^81^ A study conducted in eastern Spain found higher weed‐seed removal in no‐till fields compared to conventional fields. Nest densities of harvester ants (*Messor barbarus*) were significantly higher in undisturbed soil.^82^ Similarly, in Central Spain, annual moldboard plowing completely eliminated the harvester ant *M. capitatus* from agricultural fields, highlighting how intensive tillage operations can exclude these important seed predators.^83^ Predatory ants may also benefit from reduced disturbance and provide stronger biological control in no‐till fields.^84^ For example, in Brazil, no‐till systems had nearly twice as many ant species (16 vs 9) as conventionally tilled systems. Predation also increased in no‐till plots, suggesting that ants may exert stronger biological control in no‐till systems.^84^ Physical changes that ants make to soil structure through nest construction also compound over time in no‐till systems, potentially enhancing soil porosity, water infiltration, and organic matter incorporation to a greater degree than in periodically disturbed soils.^8^, ^85^ Together, these findings suggest that long‐term no‐till management of soil may strengthen ecological roles of ants in agricultural systems. However, long‐term experiments must test the hypothesis that extended periods of no‐till enhance pest suppression, weed‐seed removal, and soil improvement by ants.

### 2.2 Benefits of cover crops for ant communities

Beyond the advantages of no‐till systems, cover crops represent another conservation practice gaining popularity that may significantly benefit ant populations and communities. Integrating cover crops into cash‐crop rotations can provide multiple ecosystem services, including stabilizing soil, enhancing soil fertility, and improving control of weeds and invertebrate pests.^86^, ^87^, ^88^, ^89^ Cover crops create physical habitat and architectural diversity, and provide additional food resources that can support more diverse and abundant ant communities.^26^

Growing evidence indicates that cover crops indirectly improve control of insect pests by supporting larger natural‐enemy communities, which can often keep pest pressure below economic thresholds.^34^, ^90^, ^91^, ^92^, ^93^ Studies in orchard and vineyard systems have documented that cover crops enhance ant populations with associated benefits for pest control.^94^ For example, a study conducted in eastern Austria showed that ant densities were significantly higher in vineyard plots with cover crops compared to those left fallow, demonstrating that vegetation‐management practices can directly influence beneficial‐arthropod communities in perennial cropping systems.^95^ Recent research has begun to demonstrate similar patterns in annual field crops, with higher ant abundance observed in plots planted with cover crops compared to those left fallow.^34^ Moreover, a study conducted in southwest Michigan found that organic corn‐soy‐wheat rotations with cover crops supported higher ant diversity and provided favorable timing of ant activity for pest control.^26^ While conventional plots experienced ant foraging primarily after crop harvest, organic plots maintained moderate ant activity throughout the growing season, resulting in twice the pest suppression potential when ants were active during crop growth.^26^

Cover crops can benefit ant communities through multiple mechanisms. Increased plant biomass and diversity create more complex habitat structure, offering additional nesting sites and shelter from extreme temperatures.^26^, ^96^ Cover‐crop roots and residues also increase soil organic matter, supporting more abundant soil fauna that can serve as prey for predatory ants.^74^, ^97^, ^98^, ^99^, ^100^

Combining no‐till practices with cover crops may create particularly favorable conditions for ants in agricultural systems. While no‐till farming preserves nest structures and reduces disturbance, cover crops enhance resource availability and habitat complexity, addressing multiple factors that tend to limit ant populations.^7^, ^26^, ^32^, ^82^, ^101^, ^102^ No‐till and cover crops may strengthen ecosystem services provided by ants, including pest suppression,^26^, ^84^ weed‐seed removal,^36^, ^80^, ^82^ and soil improvement.^8^, ^43^ In some systems, ants may also increase agricultural productivity through processes such as improved water infiltration and soil nitrogen availability.^85^

### 2.3 Integrated pest management can maximize the potential of ants

IPM provides a systematic framework that decreases pest damage to crops by combining complementary tactics, including biological control, habitat manipulation, cultural practices, resistant varieties, and targeted insecticide use.^103^ IPM emphasizes prevention and monitoring, and intervention only when necessary, making it an ideal strategy for conserving ant populations and their ecosystem services in agricultural systems.^104^ For farmers seeking to harness ants for biological control, IPM may provide an approach that balances pest management with conservation of natural enemies. The success of ant conservation in agricultural systems depends on the type and timing of insecticide use. Broad‐spectrum and systemic insecticides can cause lethal and sublethal effects on ant populations.^105^, ^106^, ^107^ In contrast, threshold‐based approaches reduce insecticide use and may therefore be more compatible with ant conservation,^37^, ^38^ although support for this hypothesis in ants remains limited.

Economic thresholds reduce unnecessary insecticide applications.^103^ A recent meta‐analysis across 34 crops found that threshold‐based approaches reduced insecticide use by 44% and supported greater abundance of beneficial arthropods than calendar‐based programs.^37^ These findings suggest that such approaches may similarly benefit ant populations in agricultural systems. When insecticides become necessary, farmers should select products with minimal effects on non‐target species.^108^ Selecting insecticides with minimal effects on non‐target species can help conserve beneficial arthropods.^108^, ^109^ For example, in Australian mango orchards, populations of weaver ants remained abundant throughout the growing season when farmers used ‘soft’ chemicals (potassium soap and white oil) within an IPM framework.^110^ Similarly, in Beninese cashew orchards, weaver ants remained abundant when farmers used spot applications of GF‐120 (0.02% Spinosad) within an IPM program.^109^ Given the susceptibility of ants to insecticides,^106^, ^107^, ^111^ selective products may be particularly beneficial for ant conservation in agricultural systems. Farmers may benefit when they conserve ant populations. In some agricultural systems, ants can suppress pests as effectively as chemical insecticides.^112^ For example, in cashew plantations in Australia, weaver ants suppressed pest populations more effectively than chemical insecticides.^112^

Unfortunately, while using less insecticide and softer products would help conserve ant communities, large acreage crops in the US are currently being treated more frequently with more toxic insecticides than in previous eras.^113^, ^114^, ^115^, ^116^ Insecticidal seed treatments, along with soil‐applied and foliar‐applied insecticides, reduce ant abundance and diversity throughout growing season.^105^, ^106^, ^111^ Applications of preventative insecticides, especially early‐season treatments, can reduce ant populations before they establish effective colonies, thereby disrupting their potential for season‐long pest suppression.^106^ To gain more benefits from ants (and other natural enemies), researchers, extension personnel, and farmers will need to strive to avoid unnecessary insecticide use; IPM provides the best framework to do so.^37^, ^103^, ^117^, ^118^

An integrated approach that combines IPM, cover crops, and no‐till management may create conditions that support ant communities. Whether these communities provide meaningful biological control in cropping systems in temperate regions remains unclear and requires further study

## 3 FUTURE DIRECTIONS

### 3.1 Methodological advances needed

Standardized protocols for ant sampling using pitfall traps and baits are well established, as are sentinel prey assays and seed cards for assessing functional roles.^119^, ^120^, ^121^, ^122^, ^123^ However, significant methodological improvements are still needed to advance our understanding of ecosystem services provided by ants in no‐till agricultural systems.^10^, ^26^, ^119^, ^124^, ^125^, ^126^, ^127^ Challenges lie not in developing new sampling methods, but in adapting existing protocols to agricultural contexts. This requires integrating multiple techniques to provide comprehensive assessments of ecosystem services provided by ants. Molecular gut content analysis represents a particularly promising but underutilized approach for assessing ant predation in agricultural settings.^128^ However, application of this technique to ants and other ground‐dwelling arthropods has been limited due to PCR inhibition challenges when amplifying prey DNA from ant gut contents.^129^, ^130^, ^131^ Recent advances have identified that inhibition primarily occurs in the ant gaster, particularly within the foregut and crop structures, with significant variation among species.^129^, ^130^ Effective protocols now exist to overcome these limitations, including exuding crop contents onto UV‐sterilized tissue before DNA extraction and adding bovine serum albumin to PCR reagents when inhibition occurs.^130^

Stable‐isotope analysis offers another powerful tool for understanding trophic ecology of ants in agricultural systems, yet its application in temperate no‐till farms remains virtually unexplored. This technique can reveal trophic relationships and dietary shifts that are difficult to detect through direct observation, particularly for cryptic predation that occurs belowground or nocturnally.^132^, ^133^, ^134^, ^135^ Studies using stable‐isotope analysis have demonstrated that invasive ants can shift their trophic position following establishment, moving from primarily carnivorous to more omnivorous feeding strategies as they exploit different resource bases.^136^ Recent studies have demonstrated how omnivorous ants adjust their trophic position and macronutrient preferences in response to habitat changes, showing reduced carnivory and increased protein limitation in resource‐poor environments.^134^ Recent applications in grassland systems have shown how disturbance affects ant trophic structure, with ants in disturbed areas feeding at lower trophic levels.^137^ Using similar approaches in no‐till fields could shed light on the role of ants in no‐till fields as different resources become available.

### 3.2 Management‐oriented research

Integrating natural‐enemy conservation into existing IPM programs represents an important opportunity to improve sustainable pest management in no‐till systems. Ants form part of a larger natural‐enemy community, and future studies should determine whether practices that conserve ants also benefit other beneficial arthropods. Research should focus on developing practical recommendations that farmers can implement without major changes to their operations. Another priority is to evaluate whether diverse communities of natural enemies provide more robust pest control than individual species in agricultural systems.^138^ Researchers should also investigate how cover‐crop selection and management can support natural‐enemy communities, including ants, while meeting other agricultural objectives. Different cover‐crop species may provide habitat and food resources of varying quality for beneficial arthropods, but these relationships remain poorly understood in temperate cropping systems. Additional work should examine how cover‐crop mixtures, planting dates, and termination methods influence natural‐enemy abundance and pest control. Cover‐crop recommendations that simultaneously support soil health, weed suppression, and populations of beneficial arthropods would provide valuable tools for integrated farm management.^86^, ^87^, ^92^, ^139^

Economic analysis should quantify ecosystem services provided by natural‐enemy communities in no‐till systems to demonstrate their value to farmers. These analyses should estimate the monetary value of pest suppression, weed‐seed removal, and soil improvement provided by ants and other natural enemies, and compare these benefits with the costs of implementing conservation practices. Such economic assessments could provide evidence to support policy incentives for practices that enhance natural‐enemy communities and their ecosystem services.

Farmers need decision support tools that help them integrate natural‐enemy conservation into pest‐management planning. These tools could provide guidance on when and how to modify management practices to support beneficial arthropods, how to monitor their activity, associated pest suppression, and how to balance conservation with other agricultural priorities. Web‐based platforms that integrate data on pest populations, current weather conditions, and management advice could help farmers incorporate biological control into their IPM programs.^140^ Researchers should also develop user‐friendly interfaces and validate these tools across diverse farming operations to ensure their practical utility and adoption.

### 3.3 Soil hydrology and biopore function

Ants create biopores that influence soil hydrology.^141^ As climate change alters rainfall patterns, studies should investigate whether biopore networks created by ants can help agricultural systems withstand drought and extreme rainfall.^142^, ^143^ Through nest construction, ants alter biopore density, depth, and spatial arrangement, which affects water infiltration and drainage.^143^, ^144^, ^145^ Ant nest entrances can increase infiltration rates by more than 20‐fold relative to areas without nests.^146^ Field experiments also show that ants and termites increased wheat yield by 36% by improving water infiltration through tunnel networks.^85^ These water‐management benefits may be particularly important under extreme precipitation. In no‐till systems, low bulk density of soil can reduce yield by approximately 10% because poor drainage limits water movement.^147^ Future studies should test whether ant‐created biopores improve drainage capacity and reduce yield losses. To address this question, studies should use three‐dimensional models to capture differences in biopore structure among ant species and predict how ant community composition shapes movement of soil water in agricultural systems. This approach would clarify whether conserving diverse ant communities improves crop tolerance to water stress beyond their roles in pest suppression and soil fertility.

### 3.4 Research priorities for ant ecology and function

Several fundamental knowledge gaps must be addressed to fully understand and optimize functional roles of ants in temperate, no‐till agricultural systems. First, it is necessary to document community composition of ant species among no‐till farming systems in different temperate regions. We currently have a limited understanding of the ant species that occur in fields of temperate row crops, their relative abundances, and how ant communities respond to different management practices. Such baseline information will establish which species have the greatest potential for pest suppression and other ecosystem services, and for developing management recommendations that support populations of those ant taxa.

Second, we must determine activity patterns for ant species throughout growing seasons. Understanding when ant colonies forage most intensively, how their behavior changes in response to prey availability, and how these patterns align with vulnerable periods in life cycles of pests could inform the timing of management decisions. Cover crops, crop rotations, and other conservation practices likely influence these temporal patterns in ways that could help farmers maximize biological control services while maintaining agricultural productivity.

Third, quantifying the impact of ant predation on economically important pest species is another critical research priority. While studies have documented ants preying on various agricultural pests, few have quantified the actual reduction in pest damage or crop losses attributable to ant activity, especially in temperate annual crops.^26^ More studies need to manipulate ant activity and assess functional roles using sentinel prey and seed cards to determine whether ant predation can meaningfully reduce pest populations below economic thresholds in temperate crop systems. Such studies should focus on ground and soil‐dwelling pest stages where ant predation may be most effective, including cutworm larvae, wireworms, slugs, and overwintering life stages of key pests.^63^

## 4 CONCLUSION

No‐till farming systems in temperate regions present an underexploited opportunity for harnessing ant‐mediated ecosystem services in annual crop production. While tropical and subtropical agriculture has long recognized ants as valuable biological‐control agents, annual‐cropping systems in temperate regions have largely overlooked their potential contributions. This review identified several mechanisms through which ant communities can benefit no‐till farms, including pest predation, weed‐seed removal, soil improvement, and chemical deterrence of herbivores. However, quantitative evidence for these effects remains limited in temperate agricultural systems.

No‐till practices, when combined with cover cropping and IPM, may create favorable conditions for ants to establish and persist. No‐till systems preserve nest architecture and foraging network that tillage would otherwise destroy, while cover crops provide additional resources and habitat complexity. When farmers use insecticides judiciously through IPM, ant communities may maintain greater abundance and diversity, which may support meaningful ecosystem services. In regions such as the Mid‐Atlantic USA, where farmers adopt these practices, predator communities, including ants, may contribute more to pest management as conservation agriculture expands.^34^

Several research priorities emerge from this review. Future research should quantify how ant communities function in temperate no‐till systems. This effort will require linking ant abundance and diversity to measurable effects on pest suppression, soil processes, and outcomes relevant to crop management. Coordinated efforts among ecologists, agronomists, and economists should determine whether ant activity can reduce pest populations below economic thresholds under field conditions. Such research should also quantify broader value of these ecosystem services to support conservation‐oriented management and policy.

Molecular gut‐content analysis and stable‐isotope techniques now allow scientists to address previously unanswered questions about the trophic ecology of ants. These tools, combined with traditional sampling methods and exclusion experiments, may reveal cryptic roles of ants in pest management in agricultural systems. Studies should apply these methods across gradients of management intensity, from conventional tillage to long‐term no‐till, to clarify how agricultural practices influence functional role of ants.

Farmers will adopt practices that conserve ants only when they see tangible benefits. Unlike tropical systems, where single ant species provide substantial pest control, temperate systems likely require diverse assemblages working with other natural enemies to achieve effective biological control. Management recommendations must therefore consider ants as one component of IPM rather than a standalone solution. Future research should prioritize practical outcomes, including reduced insecticide use, greater water infiltration, more stable yields, and greater profitability through enhanced ecosystem services.

Agriculture faces mounting pressure to produce more food with fewer environmental impacts, making biological control increasingly important for sustainable intensification. Ants represent a poorly recognized and underutilized resource in agricultural systems in temperate regions, even though they can dominate numerically, adapt to diverse ecological conditions, and perform multiple ecological functions.^1^, ^27^, ^148^, ^149^ Farmers who adopt management practices that conserve and enhance ant communities, particularly no‐till farming combined with cover cropping and IPM, can harness these ecosystem engineers to build more resilient and sustainable systems. Ecologists, agronomists, and farmers must collaborate to translate ecological knowledge into practical management strategies that benefit both agricultural yields and environmental health.

## Supporting information


**Table S1.** Ant species and ecosystem services in agricultural systems by geographic region.

## Data Availability

Data sharing not applicable to this article as no datasets were generated or analysed during the current study.

## References

[1] Hölldobler B and Wilson E , The Ants, Vol. 12, Havard University Press, Cambridge, MA p. 732 (1990).

[2] Kamaru DN , Palmer TM , Riginos C , Ford AT , Belnap J , Chira RM *et al*., Disruption of an ant‐plant mutualism shapes interactions between lions and their primary prey. Science 383:433–438 (2024). 10.1126/science.adg1464. 38271503

[3] Rocha FP , Bogar TA , Ibañez Weemaels A , Hu J , Liang M , Park C *et al*., Two sides of the same coin? Ants are ecosystem engineers and providers of ecosystem services (2024). 10.25849/MYRMECOL.NEWS_034:129.

[4] Sanders D and van Veen FJF , Ecosystem engineering and predation: the multi‐trophic impact of two ant species. J Anim Ecol 80:569–576 (2011). 10.1111/j.1365-2656.2010.01796.x. 21244419

[5] Sankovitz M , Rivera MJ , Manjarress T and Mathis KM , The role of ant‐mediated biological interactions in agroecosystems. Ann Entomol Soc Am 117:79–91 (2024). 10.1093/aesa/saae002.

[6] Wills BD and Landis DA , The role of ants in north temperate grasslands: a review. Oecologia 186:323–338 (2018). 10.1007/s00442-017-4007-0. PMC5799350 29147779

[7] Elliott‐Vidaurri LV , Rivera D , Noval A , Choudhury RA and Penn HJ , Red Harvester Ant (*Pogonomyrmex barbatus* F. Smith; hymenoptera: Formicidae) preference for cover crop seeds in South Texas. Agronomy 12:1099 (2022). 10.3390/agronomy12051099.

[8] Farji‐Brener AG and Werenkraut V , The effects of ant nests on soil fertility and plant performance: a meta‐analysis. J Anim Ecol 86:866–877 (2017). 10.1111/1365-2656.12672. 28369906

[9] Wang S , Wang H , Li J and Zhang Z , Ants can exert a diverse effect on soil carbon and nitrogen pools in a Xishuangbanna tropical forest. Soil Biol Biochem 113:45–52 (2017). 10.1016/j.soilbio.2017.05.027.

[10] Wills BD , Kim TN , Fox AF , Gratton C and Landis DA , Reducing native ant abundance decreases predation rates in Midwestern grasslands. Environ Entomol 48:1360–1368 (2019). 10.1093/ee/nvz127. PMC6894410 31713603

[11] Way MJ and Khoo KC , Role of ants in pest management. Annu Rev Entomol 37:479–503 (1992). 10.1146/annurev.en.37.010192.002403.

[12] Huang H and Yang P , The ancient cultured citrus ant. Bioscience 37:665–671 (1987).

[13] van Mele P , A historical review of research on the weaver ant Oecophylla in biological control. Agric For Entomol 10:13–22 (2008). 10.1111/j.1461-9563.2007.00350.x.

[14] Gonthier DJ , Pardee GL and Philpott SM , Azteca instabilis ants and the defence of a coffee shade tree: an ant–plant association without mutual rewards in Chiapas, Mexico. J Trop Ecol 26:343–346 (2010). 10.1017/S0266467409990666.

[15] Perfecto I and Vandermeer J , The effect of an ant‐hemipteran mutualism on the coffee berry borer (*Hypothenemus hampei*) in southern Mexico. Agric Ecosyst Environ 117:218–221 (2006). 10.1016/j.agee.2006.04.007.

[16] Philpott SM and Armbrecht I , Biodiversity in tropical agroforests and the ecological role of ants and ant diversity in predatory function. Ecol Entomol 31:369–377 (2006). 10.1111/j.1365-2311.2006.00793.x.

[17] Thurman JH , Northfield TD and Snyder WE , Weaver ants provide ecosystem services to tropical tree crops. Front Ecol Evol 7:120 (2019). 10.3389/fevo.2019.00120.

[18] Eubanks MD , Estimates of the direct and indirect effects of red imported fire ants on biological control in field crops. Biol Control 21:35–43 (2001). 10.1006/bcon.2001.0923.

[19] Way M , Islam Z , Heong K and Joshi R , Ants in tropical irrigated rice: distribution and abundance, especially of *Solenopsis geminata* (hymenoptera: Formicidae). Bull Entomol Res 88:467–476 (1998). 10.1017/S0007485300042218.

[20] Whitcomb WH , Denmark HA , Bhatkar AP and Greene GL , Preliminary studies on the ants of Florida soybean fields. Fla Entomol 55:129 (1972). 10.2307/3493137.

[21] Seagraves MP and McPherson RM , Monitoring red imported fire ant (Hymenoptera: Formicidae) foraging behavior and impact on foliage‐dwelling arthropods on soybean produced under three ant suppression regimes. J Entomol Sci 41:374–384 (2006). 10.18474/0749-8004-41.4.374.

[22] Ivens ABF and Kronauer DJC , Aphid‐farming ants. Curr Biol 32:R813–R817 (2022). 10.1016/j.cub.2022.06.072. 35944477

[23] Kaplan I and Eubanks MD , Aphids Alter the community‐wide impact of fire ants. Ecology 86:1640–1649 (2005). 10.1890/04-0016.

[24] Styrsky JD and Eubanks MD , A facultative mutualism between aphids and an invasive ant increases plant reproduction. Ecol Entomol 35:190–199 (2010). 10.1111/j.1365-2311.2009.01172.x.

[25] Wu D , Zeng L , Lu Y and Xu Y , Effects of *Solenopsis invicta* (hymenoptera: Formicidae) and its interaction with aphids on the seed productions of mungbean and rapeseed plants. J Econ Entomol 107:1758–1764 (2014). 10.1603/EC14162. 26309264

[26] Helms JA IV , Smith J , Clark S , Knupp K and Haddad NM , Ant communities and ecosystem services in organic versus conventional agriculture in the US Corn Belt. Environ Entomol 50:1276–1285 (2021). 10.1093/ee/nvab105. 34554252

[27] Schultheiss P , Nooten SS , Wang R , Wong MKL , Brassard F and Guénard B , The abundance, biomass, and distribution of ants on earth. Proc Natl Acad Sci USA 119:e2201550119 (2022). 10.1073/pnas.2201550119. PMC9546634 36122199

[28] Dunn RR , Agosti D , Andersen AN , Arnan X , Bruhl CA , Cerdá X *et al*., Climatic drivers of hemispheric asymmetry in global patterns of ant species richness. Ecol Lett 12:324–333 (2009). 10.1111/j.1461-0248.2009.01291.x. 19292793

[29] Offenberg J , REVIEW: ants as tools in sustainable agriculture. J Appl Ecol 52:1197–1205 (2015). 10.1111/1365-2664.12496.

[30] Schifani E , Giannetti D and Grasso DA , Ant‐mediated biological control: a global meta‐synthesis of antagonistic interactions against pests. J Pest Sci 99:36 (2026). 10.1007/s10340-025-01970-7.

[31] Perfecto I , Dynamics of *Solenopsis geminata* in a tropical fallow field after ploughing. Oikos 62:139–144 (1991). 10.2307/3545258.

[32] Robertson LN , Kettle BA and Simpson GB , The influence of tillage practices on soil macrofauna in a semi‐arid agroecosystem in northeastern Australia. Agric Ecosyst Environ 48:149–156 (1994). 10.1016/0167-8809(94)90085-X.

[33] Myers R and Joseph L , Progress report: Adoption of soil health systems based on data from the 2017 U.S. Census of Agriculture—Metrics include cover crop and no‐till production practices. Soil Health Institute, 2019 (2019). Available: https://soilhealthinstitute.org.

[34] Rowen EK , Pearsons KA , Smith RG , Wickings K and Tooker JF , Early‐season plant cover supports more effective pest control than insecticide applications. Ecol Appl 32:e2598 (2022). 10.1002/eap.2598. 35343024

[35] Peck SL , Mcquaid B and Campbell CL , Using ant species (Hymenoptera: Formicidae) as a biological indicator of agroecosystem condition. Environ Entomol 27:1102–1110 (1998). 10.1093/ee/27.5.1102.

[36] Fernandes WD , Lange D , Pereira JM and Raizer J , Ant community in neotropical agrosystems: a four‐year study in conventional and no‐tillage systems. Sociobiology 65:2 (2018). 10.13102/sociobiology.v65i2.1204.

[37] Leach A , Gomez AA and Kaplan I , Threshold‐based management reduces insecticide use by 44% without compromising pest control or crop yield. Commun Earth Environ 6:710 (2025). 10.1038/s43247-025-02643-0.

[38] Löhr B and Oswald S , An integrated pest management approach towards the control of the low‐density coconut pest, *Pseudotheraptus wayi* Brown (Hemiptera: Coreidae) (1990).

[39] Drummond F and Choate B , Ants as biological control agents in agricultural cropping systems. Terr Arthropod Rev 4:157–180 (2011). 10.1163/187498311X571979.

[40] Schowalter TD and Ring DR , Biology and management of the Texas leafcutting ant (Hymenoptera: Formicidae). J Integr Pest Manag 8:16 (2017). 10.1093/jipm/pmx013.

[41] Folgarait PJ , Ant biodiversity and its relationship to ecosystem functioning: a review. Biodivers Conserv 7:1221–1244 (1998). 10.1023/A:1008891901953.

[42] MacMahon JA , Mull JF and Crist TO , Harvester ants (*Pogonomyrmex* spp.): their community and ecosystem influences. Annu Rev Ecol Evol Syst 31:265–291 (2000). 10.1146/annurev.ecolsys.31.1.265.

[43] Wu D , Du E , Eisenhauer N , Mathieu J and Chu C , Global engineering effects of soil invertebrates on ecosystem functions. Nature 640:120–129 (2025). 10.1038/s41586-025-08594-y. 39939777

[44] Carroll CR and Janzen DH , Ecology of foraging by ants. Annu Rev Ecol Evol Syst 4:231–257 (1973). 10.1146/annurev.es.04.110173.001311.

[45] Gibb H and Cunningham SA , Habitat contrasts reveal a shift in the trophic position of ant assemblages. J Anim Ecol 80:119–127 (2011). 10.1111/j.1365-2656.2010.01747.x. 20831728

[46] Anjos DV , Tena A , Viana‐Junior AB , Carvalho RL , Torezan‐Silingardi H , Del‐Claro K *et al*., The effects of ants on pest control: a meta‐analysis. Proc R Soc B 289:20221316 (2022). 10.1098/rspb.2022.1316. PMC9382213 35975443

[47] Kautz T , Research on subsoil biopores and their functions in organically managed soils: a review. Renew Agric Food Syst 30:318–327 (2015). 10.1017/S1742170513000549.

[48] Tschinkel WR and Seal JN , Bioturbation by the fungus‐gardening ant, *Trachymyrmex septentrionalis* . PLoS One 11:e0158920 (2016). 10.1371/journal.pone.0158920. PMC4938500 27391485

[49] Wuest SB , Soil biopore estimation: effects of tillage, nitrogen, and photographic resolution. Soil Tillage Res 62:111–116 (2001). 10.1016/S0167-1987(01)00218-5.

[50] Dauber J , Niechoj R , Baltruschat H and Wolters V , Soil engineering ants increase grass root arbuscular mycorrhizal colonization. Biol Fertil Soils 44:791–796 (2008). 10.1007/s00374-008-0283-5.

[51] Frouz J , Holec M and Kalčík J , The effect of *Lasius niger* (Hymenoptera, Formicidae) ant nest on selected soil chemical properties. Pedobiologia 47:205–212 (2003). 10.1078/0031-4056-00184.

[52] Frouz J and Jilková V , The effect of ants on soil properties and processes (Hymenoptera: Formicidae) (2008). .

[53] Lindström S , Timonen S , Sundström L and Johansson H , Ants reign over a distinct microbiome in forest soil. Soil Biol Biochem 139:107529 (2019). 10.1016/j.soilbio.2019.107529.

[54] Lindström S , Timonen S and Sundström L , The bacterial and fungal community composition in time and space in the nest mounds of the ant *Formica exsecta* (hymenoptera: Formicidae). Microbiolopen 10:e1201 (2021). 10.1002/mbo3.1201. PMC8289489 34459553

[55] Jackson DE and Ratnieks FLW , Communication in ants. Curr Biol 16:R570–R574 (2006). 10.1016/j.cub.2006.07.015. 16890508

[56] Traniello JFA , Foraging strategies of ants. Annu Rev Entomol 34:191–210 (1989). 10.1146/annurev.en.34.010189.001203.

[57] Adandonon A , Vayssières J , Sinzogan A and Van Mele P , Density of pheromone sources of the weaver ant *Oecophylla longinoda* (Hymenoptera Formicidae) affects oviposition behaviour and damage by mango fruit flies (Diptera Tephritidae). Int J Pest Manag 55:285–292 (2009).

[58] Gonthier DJ , Do herbivores eavesdrop on ant chemical communication to avoid predation? PLoS One 7:e28703 (2012). 10.1371/journal.pone.0028703. PMC3250387 22235248

[59] Offenberg J , Nielsen MG , MacIntosh DJ , Havanon S and Aksornkoae S , Evidence that insect herbivores are deterred by ant pheromones. Proc R Soc Lond B 271:S433–S435 (2004). 10.1098/rsbl.2004.0210. PMC1810093 15801596

[60] Van Mele P , Vayssieres JF , Adandonon A and Sinzogan A , Ant cues affect the oviposition behaviour of fruit flies (Diptera: Tephritidae) in Africa. Physiol Entomol 34:256–261 (2009). 10.1111/j.1365-3032.2009.00685.x.

[61] Otsuki H and Yano S , Functionally different predators break down antipredator defenses of spider mites. Entomol Exp Appl 151:27–33 (2014). 10.1111/eea.12164.

[62] Kempraj V , Park SJ , Cameron DNS and Taylor PW , 1‐Octanol emitted by *Oecophylla smaragdina* weaver ants repels and deters oviposition in Queensland fruit fly. Sci Rep 12:15768 (2022). 10.1038/s41598-022-20102-0. PMC9492660 36130986

[63] López R and Potter DA , Ant predation on eggs and larvae of the black cutworm (Lepidoptera: Noctuidae) and Japanese beetle (Coleoptera: Scarabaeidae) in Turfgrass. Environ Entomol 29:116–125 (2000). 10.1603/0046-225X-29.1.116.

[64] Lal R , Reicosky DC and Hanson JD , Evolution of the plow over 10,000 years and the rationale for no‐till farming. Soil Tillage Res 93:1–12 (2007). 10.1016/j.still.2006.11.004.

[65] Bàrberi P and Lo Cascio B , Long‐term tillage and crop rotation effects on weed seedbank size and composition. Weed Res 41:325–340 (2001). 10.1046/j.1365-3180.2001.00241.x.

[66] Bockus WW and Shroyer JP , The impact of reduced tillage on SOILBORNE plant pathogens. Annu Rev Phytopathol 36:485–500 (1998). 10.1146/annurev.phyto.36.1.485. 15012510

[67] Douglas MR and Tooker JF , Slug (Mollusca: Agriolimacidae, Arionidae) ecology and management in no‐till field crops, with an emphasis on the mid‐Atlantic region. J Integr Pest Manag 3:C1–C9 (2012). 10.1603/IPM11023.

[68] Stone JA , Vyn TJ , Martin HD and Groenevelt PH , Ridge‐tillage and early‐season soil moisture and temperature on a poorly drained soil. Can J Soil Sci 69:181–186 (1989). 10.4141/cjss89-018.

[69] van Wijk WR , Larson WE and Burrows WC , Soil temperature and the early growth of corn from mulched and unmulched soil. Soil Sci Soc Am J 23:428–434 (1959). 10.2136/sssaj1959.03615995002300060020x.

[70] USDA , Soil Health Literature Summary—Effects of Conservation Practices on Soil Properties in Areas of Cropland (2015). .

[71] Kassam A , Friedrich T and Derpsch R , Global spread of conservation agriculture. Int J Environ Stud 76:29–51 (2019). 10.1080/00207233.2018.1494927.

[72] USDA . 2017. Available: https://www.nass.usda.gov/Publications/AgCensus/2017/Full_Report/Volume_1,_Chapter_1_US/usv1.pdf

[73] Welsh DS and Rey M , Saving the Chesapeake Bay Watershed Requires Better Coordination of Environmental and Agricultural Resources (2006). .

[74] Appenfeller LR , Brainard DC , Hayden ZD and Szendrei Z , Beneficial and pest arthropod responses to tillage and cover crop residues in organic cucurbits. Environ Entomol 51:1182–1190 (2022). 10.1093/ee/nvac076. 36178322

[75] House GJ and Stinner BR , Arthropods in no‐tillage soybean agroecosystems: community composition and ecosystem interactions. Environ Manag 7:23–28 (1983). 10.1007/BF01867037.

[76] Douglas MR , Rohr JR and Tooker JF , EDITOR’S CHOICE: neonicotinoid insecticide travels through a soil food chain, disrupting biological control of non‐target pests and decreasing soya bean yield. J Appl Ecol 52:250–260 (2015). 10.1111/1365-2664.12372.

[77] Rowen EK , Regan KH , Barbercheck ME and Tooker JF , Is tillage beneficial or detrimental for insect and slug management? A meta‐analysis. Agric Ecosyst Environ 294:106849 (2020). 10.1016/j.agee.2020.106849.

[78] Aref S and Pike DR , Midwest farmers' perceptions of crop pest infestation. Agron J 90:819–825 (1998). 10.2134/agronj1998.00021962009000060013x.

[79] Stinner BR and House GJ , Arthropods and other invertebrates in conservation‐tillage agriculture. Annu Rev Entomol 35:299–318 (1990). 10.1146/annurev.en.35.010190.001503.

[80] Schifani E , Geppert C , Giannetti D , Anfora G , Burgio G , Chiesa SG *et al*., Organic management, reduced tillage, and increased vegetation cover promote ant abundance and diversity in Mediterranean vineyards. J Insect Conserv 30:15 (2026). 10.1007/s10841-026-00747-2.

[81] Baraibar B , Torra J and Westerman PR , Harvester ant (*Messor barbarus* (L.)) density as related to soil properties, topography and management in semi‐arid cereals. Appl Soil Ecol 51:60–65 (2011). 10.1016/j.apsoil.2011.08.012.

[82] Baraibar B , Westerman PR , Carrión E and Recasens J , Effects of tillage and irrigation in cereal fields on weed seed removal by seed predators. J Appl Ecol 46:380–387 (2009). 10.1111/j.1365-2664.2009.01614.x.

[83] Diaz M , Spatial patterns of granivorous ant nest abundance and nest site selection in agricultural landscapes of central Spain. Insect Soc 38:351–363 (1991). 10.1007/BF01241871.

[84] Lange D , Fernandes WD , Raizer J and Faccenda O , Predacious activity of ants (Hymenoptera: Formicidae) in conventional and in no‐till agriculture systems. Braz Arch Biol Technol 51:1199–1207 (2008). 10.1590/S1516-89132008000600015.

[85] Evans TA , Dawes TZ , Ward PR and Lo N , Ants and termites increase crop yield in a dry climate. Nat Commun 2:262 (2011). 10.1038/ncomms1257. PMC3072065 21448161

[86] Schipanski ME , Barbercheck M , Douglas MR , Finney DM , Haider K , Kaye JP *et al*., A framework for evaluating ecosystem services provided by cover crops in agroecosystems. Agric Syst 125:12–22 (2014). 10.1016/j.agsy.2013.11.004.

[87] Koch RL , Sezen Z , Porter PM , Ragsdale DW , Wyckhuys KAG and Heimpel GE , On‐farm evaluation of a fall‐seeded rye cover crop for suppression of soybean aphid (Hemiptera: Aphididae) on soybean. Agric For Entomol 17:239–246 (2015). 10.1111/afe.12099.

[88] Koudahe K , Allen SC and Djaman K , Critical review of the impact of cover crops on soil properties. Int Soil Water Conserv Res 10:343–354 (2022). 10.1016/j.iswcr.2022.03.003.

[89] Fajemisin A , Kaur S , Vasquez A , Racelis A and Kariyat R , Do cover crops influence beneficial and herbivorous arthropod communities across growing seasons? J Pest Sci 99:92 (2026). 10.1007/s10340-026-02051-z.

[90] Bowers C , Toews M , Liu Y and Schmidt JM , Cover crops improve early season natural enemy recruitment and pest management in cotton production. Biol Control 141:104149 (2020). 10.1016/j.biocontrol.2019.104149.

[91] Le Gall M and Tooker JF , Developing ecologically based pest management programs for terrestrial molluscs in field and forage crops. J Pest Sci 90:825–838 (2017). 10.1007/s10340-017-0858-8.

[92] Lundgren JG and Fergen JK , Enhancing predation of a subterranean insect pest: a conservation benefit of winter vegetation in agroecosystems. Appl Soil Ecol 51:9–16 (2011). 10.1016/j.apsoil.2011.08.005.

[93] Witmer JE , Hough‐Goldstein JA and Pesek JD , Ground‐dwelling and foliar arthropods in four cropping systems. Environ Entomol 32:366–376 (2003). 10.1603/0046-225X-32.2.366.

[94] Altieri MA and Schmidt LL , The dynamics of colonizing arthropod communities at the interface of abandoned, organic and commercial apple orchards and adjacent woodland habitats. Agric Ecosyst Environ 16:29–43 (1986). 10.1016/0167-8809(86)90073-3.

[95] Reiff JM , Kolb S , Entling MH , Herndl T , Möth S , Walzer A *et al*., Organic farming and cover‐crop management reduce pest predation in Austrian vineyards. Insects 12:3 (2021). 10.3390/insects12030220. PMC7999927 33806420

[96] Haan NL , Helms JA and Landis DA , Bioenergy cropping systems shape ant community composition and functional roles. Front Conserv Sci 4:1283225 (2024). 10.3389/fcosc.2023.1283225.

[97] Aldebron C , Jones MS , Snyder WE and Blubaugh CK , Soil organic matter links organic farming to enhanced predator evenness. Biol Control 146:104278 (2020). 10.1016/j.biocontrol.2020.104278.

[98] Ding G , Liu X , Herbert S , Novak J , Amarasiriwardena D and Xing B , Effect of cover crop management on soil organic matter. Geoderma 130:229–239 (2006). 10.1016/j.geoderma.2005.01.019.

[99] Duyck PF , Lavigne A , Vinatier F , Achard R , Okolle JN and Tixier P , Addition of a new resource in agroecosystems: do cover crops alter the trophic positions of generalist predators? Basic Appl Ecol 12:47–55 (2011). 10.1016/j.baae.2010.11.009.

[100] Tillman G , Schomberg H , Phatak S , Mullinix B , Lachnicht S , Timper P *et al*., Influence of cover crops on insect pests and predators in conservation tillage cotton. J Econ Entomol 97:1217–1232 (2004). 10.1093/jee/97.4.1217. 15384330

[101] Sharley DJ , Hoffmann AA and Thomson LJ , The effects of soil tillage on beneficial invertebrates within the vineyard. Agric For Entomol 10:233–243 (2008). 10.1111/j.1461-9563.2008.00376.x.

[102] Hahn D , Sallenave R , Pornaro C and Leinauer B , Managing cool‐season turfgrass without herbicides: optimizing maintenance practices to control weeds. Crop Sci 60:2204–2220 (2020). 10.1002/csc2.20175.

[103] Stern VM , Smith RF , van den Bosch K and Ragen KS , The integration of chemical and biological control of the spotted alfalfa aphid: the integrated control concept. Hilgardia 29:81–101 (1959).

[104] Angon PB , Mondal S , Jahan I , Datto M , Antu UB , Ayshi FJ *et al*., Integrated pest management (IPM) in agriculture and its role in maintaining ecological balance and biodiversity. Adv Agric 2023:5546373 (2023). 10.1155/2023/5546373.

[105] Perfecto I , Indirect and direct effects in a tropical agroecosystem: the maize‐pest‐ant system in Nicaragua. Ecology 71:2125–2134 (1990). 10.2307/1938626.

[106] Schläppi D , Kettler N , Straub L , Glauser G and Neumann P , Long‐term effects of neonicotinoid insecticides on ants. Commun Biol 3:1–9 (2020). 10.1038/s42003-020-1066-2. PMC7320190 32591582

[107] Schläppi D , Al‐Hashemi A , Wasif V , Masson F , Leckie L and Stroeymeyt N , Synergistic effect of the next generation insecticide flupyradifurone with a fungal pathogen in the ant Lasius Niger. Sci Rep 15:36636 (2025). 10.1038/s41598-025-20393-z. PMC12541035 41120407

[108] Torres JB and Bueno A d F , Conservation biological control using selective insecticides – a valuable tool for IPM. Biol Control 126:53–64 (2018). 10.1016/j.biocontrol.2018.07.012.

[109] Anato FM , Wargui RB , Sinzogan AAC , Offenberg J , Adandonon A , Vayssières JF *et al*., Reducing losses inflicted by insect pests on cashew, using weaver ants as a biological control agent. Agric For Entomol 17:285–291 (2015). 10.1111/afe.12105.

[110] Peng R and Christian K , Integrated pest management in mango orchards in the Northern Territory Australia, using the weaver ant, *Oecophylla smaragdina*, (Hymenoptera: Formicidae) as a key element. Int J Pest Manag 51:149–155 (2005). 10.1080/09670870500131749.

[111] Disque HH , Hamby KA , Dubey A , Taylor C and Dively GP , Effects of clothianidin‐treated seed on the arthropod community in a mid‐Atlantic no‐till corn agroecosystem. Pest Manag Sci 75:969–978 (2019). 10.1002/ps.5201. 30192045

[112] Peng RK , Implementing Ant Technology in Commercial Cashew Plantations and Continuation of Transplanted Green Ant Colony Monitoring: A Report for the Rural Industries Research and Development Corporation. Rural Industries Research and Development Corp., Barton, A.C.T. (2004).

[113] Douglas MR and Tooker JF , Large‐scale deployment of seed treatments has driven rapid increase in use of neonicotinoid insecticides and preemptive Pest Management in U.S. field crops. Environ Sci Technol 49:5088–5097 (2015). 10.1021/es506141g. 25793443

[114] Tooker JF , Douglas MR and Krupke CH , Neonicotinoid seed treatments: limitations and compatibility with integrated pest management. Agric Environ Lett 2:ael2017.08.0026 (2017). 10.2134/ael2017.08.0026.

[115] Wolfram J , Bussen D , Bub S , Petschick LL , Herrmann LZ and Schulz R , Increasing applied pesticide toxicity trends counteract the global reduction target to safeguard biodiversity. Science 391:616–621 (2026). 10.1126/science.aea8602. 41643011

[116] Douglas MR , Sponsler DB , Lonsdorf EV and Grozinger CM , County‐level analysis reveals a rapidly shifting landscape of insecticide hazard to honey bees (*Apis mellifera*) on US farmland. Sci Rep 10:797 (2020). 10.1038/s41598-019-57225-w. PMC6972851 31964921

[117] Pecenka JR , Ingwell LL , Foster RE , Krupke CH and Kaplan I , IPM reduces insecticide applications by 95% while maintaining or enhancing crop yields through wild pollinator conservation. Proc Natl Acad Sci USA 118:e2108429118 (2021). 10.1073/pnas.2108429118. PMC8612243 34697238

[118] Busch AK , Douglas MR , Malcolm GM , Karsten HD and Tooker JF , A high‐diversity/IPM cropping system fosters beneficial arthropod populations, limits invertebrate pests, and produces competitive maize yields. Agric Ecosyst Environ 292:106812 (2020). 10.1016/j.agee.2019.106812.

[119] Lövei GL and Ferrante M , A review of the sentinel prey method as a way of quantifying invertebrate predation under field conditions. Insect Sci 24:528–542 (2017). 10.1111/1744-7917.12405. 27686246

[120] Underwood EC and Fisher BL , The role of ants in conservation monitoring: if, when, and how. Biol Conserv 132:166–182 (2006). 10.1016/j.biocon.2006.03.022.

[121] Westerman PR , Atanackovic V , Royo‐Esnal A and Torra J , Differential weed seed removal in dryland cereals. Arthropod Plant Interact 6:591–599 (2012). 10.1007/s11829-012-9211-6.

[122] Ferrante M and Lövei GL , The sentinel approach to quantify ecosystem function intensities. Methods Ecol Evol 16:2305–2317 (2025). 10.1111/2041-210X.70143.

[123] Iasczczaki RS , Beghelli GM , Carneiro GS and de Assis CHB , Honeydew‐producing pest enhances density and predation behavior of ants: a case study in erva‐mate agroecosystems. Arthropod Plant Interact 19:60 (2025). 10.1007/s11829-025-10169-7.

[124] Diaz R , Knutson A and Bernal JS , Effect of the red imported fire ant on cotton aphid population density and predation of bollworm and beet armyworm eggs. J Econ Entomol 97:222–229 (2004). 10.1093/jee/97.2.222. 15154439

[125] Jones VP , Westcott DM , Finson NN and Nishimoto RK , Relationship between community structure and southern green stink bug (Heteroptera: Pentatomidae) damage in macadamia nuts. Environ Entomol 30:1028–1035 (2001). 10.1603/0046-225X-30.6.1028.

[126] Olson DM and Ruberson JR , Crop‐specific mortality of southern green stink bug eggs in Bt‐ and non‐Bt cotton, soybean and peanut. Biocontrol Sci Tech 22:1417–1428 (2012). 10.1080/09583157.2012.731495.

[127] Rowen EK , Pearsons KA , Smith RG , Wickings K and Tooker JF , Insecticides may facilitate the escape of weeds from biological control. PeerJ 13:e18597 (2025). 10.7717/peerj.18597. PMC11737330 39822979

[128] Álvarez HA , García‐García A , Sandoval P , Martín‐Blázquez R , Seifert B , Tinaut A *et al*., Elucidating the trophic role of *Tapinoma ibericum* (Hymenoptera: Formicidae) as a potential predator of olive pests. J Appl Entomol 147:667–675 (2023). 10.1111/jen.13160.

[129] Gadau J , DNA isolation from ants. Cold Spring Harb Protoc 2009:pdb.prot5245 (2009). 10.1101/pdb.prot5245. 20147210

[130] Penn HJ , Chapman EG and Harwood JD , Overcoming PCR inhibition during DNA‐based gut content analysis of ants. Environ Entomol 45:1255–1261 (2016). 10.1093/ee/nvw090. 27452765

[131] Juen A and Traugott M , Amplification facilitators and multiplex PCR: tools to overcome PCR‐inhibition in DNA‐gut‐content analysis of soil‐living invertebrates. Soil Biol Biochem 38:1872–1879 (2006). 10.1016/j.soilbio.2005.11.034.

[132] Roeder KA and Kaspari M , From cryptic herbivore to predator: stable isotopes reveal consistent variability in trophic levels in an ant population. Ecology 98:297–303 (2017). 10.1002/ecy.1641. 28052342

[133] Tillberg CV , McCarthy DP , Dolezal AG and Suarez AV , Measuring the trophic ecology of ants using stable isotopes. Insect Soc 53:65–69 (2006). 10.1007/s00040-005-0836-7.

[134] Tsang TPN , Guénard B and Bonebrake TC , Omnivorous ants are less carnivorous and more protein‐limited in exotic plantations. J Anim Ecol 89:1941–1951 (2020). 10.1111/1365-2656.13249. 32379899

[135] Ottonetti L , Tucci L , Chelazzi G and Santini G , Stable isotopes analysis to assess the trophic role of ants in a Mediterranean agroecosystem. Agric For Entomol 10:29–36 (2008). 10.1111/j.1461-9563.2007.00358.x.

[136] McCue MD , Javal M , Clusella‐Trullas S , Le Roux JJ , Jackson MC , Ellis AG *et al*., Using stable isotope analysis to answer fundamental questions in invasion ecology: Progress and prospects. Methods Ecol Evol 11:196–214 (2020). 10.1111/2041-210X.13327.

[137] Kim T , Bartel S and Gratton C , Grassland harvesting alters ant community trophic structure: an isotopic study in tallgrass prairies. Ecol Evol 9:9815–9826 (2019). 10.1002/ece3.5523. PMC6745673 31534696

[138] Tylianakis JM and Romo CM , Natural enemy diversity and biological control: making sense of the context‐dependency. Basic Appl Ecol 11:657–668 (2010). 10.1016/j.baae.2010.08.005.

[139] Daryanto S , Fu B , Wang L , Jacinthe PA and Zhao W , Quantitative synthesis on the ecosystem services of cover crops. Earth Sci Rev 185:357–373 (2018). 10.1016/j.earscirev.2018.06.013.

[140] Jones VP , Brunner JF , Grove GG , Petit B , Tangren GV and Jones WE , A web‐based decision support system to enhance IPM programs in Washington tree fruit. Pest Manag Sci 66:587–595 (2010). 10.1002/ps.1913. 20127866

[141] Drager KI , Hirmas DR and Hasiotis ST , Effects of ant (*Formica subsericea*) nests on physical and hydrological properties of a fine‐textured soil. Soil Sci Soc Am J 80:364–375 (2016). 10.2136/sssaj2015.08.0300.

[142] IPCC , Climate Change 2021: The Physical Science Basis. Contribution of Working Group I to the Sixth Assessment Report of the Intergovernmental Panel on Climate Change. Cambridge University Press, Cambridge, UK (2021).

[143] de Bruyn LAL and Conacher AJ , The effect of ant biopores on water infiltration in soils in undisturbed bushland and in farmland in a semi‐arid environment. Pedobiologia 38:193–207 (1994). 10.1016/S0031-4056(24)00122-7.

[144] Benckiser G , Ants and sustainable agriculture. A review. Agron Sustain Dev 30:191–199 (2010). 10.1051/agro/2009026.

[145] Cammeraat ELH and Risch AC , The impact of ants on mineral soil properties and processes at different spatial scales. J Appl Entomol 132:285–294 (2008). 10.1111/j.1439-0418.2008.01281.x.

[146] Li TC , Shao MA , Jia YH , Jia XX , Huang LM and Gan M , Small‐scale observation on the effects of burrowing activities of ants on soil hydraulic processes. Eur J Soil Sci 70:236–244 (2019). 10.1111/ejss.12748.

[147] Hopkins JR and Bennett AE , Extreme rainfall shifts the drivers of *Zea mays* yield across a tillage‐intensity gradient. Appl Soil Ecol 216:106566 (2025). 10.1016/j.apsoil.2025.106566.

[148] Dantas A and Fonseca CR , Global biogeographical patterns of ants and their abiotic determinants. Perspect Ecol Conserv 21:237–246 (2023). 10.1016/j.pecon.2023.07.003.

[149] Gibb H , Dunn RR , Sanders NJ , Grossman BF , Photakis M , Abril S *et al*., A global database of ant species abundances. Ecology 98:883–884 (2017). 10.1002/ecy.1682. 27984661

