# Supplementary material for: The functional role of ants in no‐till cropping systems
Source: Pest Manag Sci. 2026 Jul 28;82(12):10886–95. doi: 10.1002/ps.71152 (PMC13756828; doi:10.1002/ps.71152)
Supplement: Supplementary file 1 [file PS-82-10886-s001.docx]

Supplementary Table 1. Ant Species and Ecosystem Services in Agricultural Systems by Geographic Region

## **Tropical/Subtropical**

| Location | Ant Species | Crop | Ecosystem Service | Quantified Impact | Reference |
| --- | --- | --- | --- | --- | --- |
| Australia (Northern Territory) | *Oecophylla smaragdina* | Mango | Pest control | *O. smaragdina* reduced seed-weevil damage to less than 1%, compared with 1.6-2.5% in plots without ants | ^1^ |
| Australia (Northern Territory) | *O. smaragdina* | Mango | Pest control | *O. smaragdina* reduced thrips damage by 3.2% on floral shoots and 10.9% on foliar shoots. | ^2^ |
| Benin | *O. longinoda* | Cashew | Pest control | *O. longinoda* increased nut yield by 78% in control plots, 122% in ant-sugar plots, and 151% under IPM | ^3^ |
| Mexico (Chiapas) | *Azteca sericeasur* | Coffee | Pest control | *A. sericeasur* suppressed coffee berry borer infestation across a wide range of pest densities | ^4^ |
| Benin | *O. longinoda* | Mango | Pest control | Fruits exposed to *O. longinoda* had fewer puparia, while fruit unexposed fruit contained eight times more puparia | ^5^ |
| India (Uttar Pradesh) | *Pheidole sp.* | Cauliflower | Pest control | *Pheidole sp* reduced leaf damaged by black cutworm larvae | ^6^ |
| Trinidad and Tobago | *Azteca sp.* | Citrus | Pest control | *Azteca sp.* prevented leaf defoliation by *Atta cephalotes* | ^7^ |
| Cameroon | *Wasmania auropunctata* | Cocoa | Pest control | *W. auropunctata* reduced cacao mirid populations | ^8^ |
| Mexico | *Pheidole spp., Solenopsis spp.* | Cotton | Pest control | Ants reduced cotton leafworm density | ^9^ |
| Philippines | *S. geminata* | Rice | Pest control | *S. geminata* reduced Channeled applesnail egg masses | ^10^ |
| South China | *S. invicta* | Banana | Pest control | *S. invicta* reduced banana skipper and banana lacebug population | ^11^ |
| Cameroon | *O. longinoda* | Cacao | Pest control | *O. longinoda* reduced mirid populations in cacao | ^12^ |
| Tanzania | *O. longinoda* | Cashew | Pest control | *O. longinoda* reduced pest damage on cashew trees | ^13^ |
| Ghana | *O. longinoda* | Cashew | Pest control | *O. longinoda* reduced pest damage on cashew trees | ^14^ |
| Tanzania | *O. longinoda* | Cashew | Pest control | Cashew trees colonized by *O. longinoda* had the lowest nut and leaf damage | ^15^ |
| Australia (Northern Territory) | *O. smaragdina* | Cashew | Pest control | *O. smaragdina* reduced pest populations, and trees with ant colonies produced higher-quality nuts | ^16^ |
| Ghana | *O. longinoda* | Citrus | Pest control | *O. longinoda* colonized citrus trees had lower fruit-fly infestation | ^17^ |
| Tanzania (Zanzibar, island of Unguja) | *O. longinoda* | Coconut | Pest control | Coconut palms with stable *O. longinoda* colonies experienced less pest damage | ^18^ |
| Mexico | *A. Instabilis* & *P. synanthropica* | Coffee | Pest control | Plants with ants had lower berry damage than plants without ants | ^19^ |
| Mexico | *A. instabilis* | Coffee | Pest control | *A. instabilis* reduced berry damage in coffee plants | ^20^ |
| Mexico | *A. sericeasur* | Coffee | Pest control | Coffee berry borer infestation was 71-82% lower on ant-protected branches | ^21^ |
| Mexico | *A. sericeasur* | Coffee | Pest control | *A. sericeasur* reduced coffee berry borer damage | ^22^ |
| USA (southeastern US) | *S. invicta* | Cotton; Soybean | Pest control | *S. invicta* abundance was negatively associated with nearly all herbivore pests in cotton and soybean | ^23^ |
| India | *Camponotus compressus* | Sponge | Pest control | *C. compressus* deterred major herbivores from feeding on plant structures | ^24^ |
| Uganda | *Lepisiota spp.* & *Myrmicaria spp.* | Maize | Pest control | Ants suppressed termite attacks on maize | ^25^ |
| USA (north-central Texas) | *S. invicta* | Maize | Pest control | Higher ant abundance corresponded with fewer corn earworm pupae in soil | ^26^ |
| Tanzania | *O. longinoda* | Mango | Pest control | *O. longinoda* reduced fruit fly infestation in mango | ^27^ |
| Tanzania | *O. longinoda* | Mango | Pest control | *O. longinoda* reduced mango seed-weevil infestation | ^28^ |
| Tanzania | *O. longinoda* | Mango | Pest control | *O. longinoda* activity reduced fruit fly damage by repelling oviposition adults | ^29^ |
| Nigeria | *O. longinoda*, *Camponotus spp.* & *Crematogaster spp.* | Cacao | Pest control | Ants reduced mirid populations in cacao | ^30^ |
| Nicaragua | Multiple ant species | Maize | Pest control | Ants suppress fall armyworm by preying on pupae | ^31^ |
| Alabama | *S. invicta* | Cotton | Pest control | *S. invicta* reduced caterpillar herbivory and increased cotton yield | ^32^ |
| Cameroon | *Crematogaster spp*., *C. brutus*, *O. longinoda* | Cacao | Pest control | Ants reduced damage by *Salhbergella singularis* and *Characoma stictigrapta* | ^33^ |
| USA (Florida) | *S. invicta* | Soybean | Pest control | *S. invicta* consumed up to 25% of small velvetbean caterpillar (*Anticarsia gemmatalis*) larvae per day | ^34^ |
| Brazil | *P. oliveirai* | Cotton | Pest control | Foraging ants attacked and removed 20% of boll weevil (*Anthonomus grandis*) adults, and *P. oliveirai* | ^35^ |
| USA (Texas) | *S. invicta* | Cotton | Pest control | *S. invicta* consumed up to 85% of boll weevil (*Anthonomus grandis*) larvae | ^36^ |
| Indonesia | *Dolichoderus spp., S. geminata* | Soybean | Pest Control | Ants preyed on eggs of pod-sucking bugs (*Nezera viridula* and *Piezodorus hybneri*) in soybean fields | ^37^ |
| Philippines | *S. geminata* | Rice | Pest control | *S. geminata* preyed on brown planthopper (*Nilaparvata lugens*) and caused high mortality of rice leaffolders (97%) | ^38^ |
| Colombia | Multiple ant species | Coffee | Pest control | Ant removed coffee berry borers (*Hypothenemus hampei*) from shaded coffee plantations | ^39^ |
| Brazil | *S. saevissima*, *Crematogaster spp*., *Pheidole spp*. | Sugarcane | Pest control | Ants attacked immature stages of sugarcane borer (*Diatraea saccharalis*) in sugarcane fields with retained straw | ^40^ |
| Martinique | *S. geminata* | Banana | Pest control | *S. geminata* preyed on 7.8-70.3% of banana weevil (*Cosmopolites sordidus*) eggs | ^41^ |
| Mexico | Multiple ant species | Coffee | Pest control | Ants reduced colonization of coffee berries by coffee berry borer |  |
| Malaysia | *O. smaragdina* | Oil palm | Pest control | *O. smaragdina* reduced bagworm (*Pteroma pendula*) abundance and foliar injury in oil palm | ^42^ |
| Indonesia | Multiple ant species | Corn | Pest control | Ants preyed on 38-50% of Asian corn borer (*Ostrinia furnaculis*) larvae and 60-64% of pupae within 14 h | ^43^ |
| Brazil | Multiple ant species | Corn | Pest control | Ant reduced abundance of southern corn rootworm (*Diabrotica speciosa*) on corn plants | ^44^ |
| Ghana | *P. megacephala* | Corn | Pest control | *P. megacephala* was the most abundant and widely distributed predator associated with fall armyworm (*Spodoptera frugiperda*) | ^45^ |
| Burkina Faso | *P. megacephala* | Corn | Pest control | *P. megacephala* was recorded as a predators associated with fall armyworm (*S. frugiperda*) | ^46^ |
| USA (Georgia) | *S. invicta* | Soybean | Pest control | *S. invicta* reduced survival of soybean looper (*Pseudoplusia includens*) and corn earworm (*Helicoverpa zea*) eggs | ^47^ |
| Tanzania | *O. longinoda* | Cashew | Pest control | Palms colonized by *O. longinoda* produced approximately twice as many coconuts as ant-free palms. | ^48^ |
| Australia (Northern Territory) | *O. smaragdina* | Mango | Pest control | *O. smaragdina* reduced damage by mango leafhopper (*Idioscopus nitidulus*) on mango shoots | ^49^ |
| Senegal | *O. longinoda* | Mango | Pest control | *O. longinoda* reduced mango infestation by fruit fly (*Bactrocera dorsalis*) and decreased pupal development | ^50^ |
| Réunion | *P. megacephala* | Sugarcane | Pest control | *P. megacephala* removed up to 82% of sugarcane stem borer (*Chilo sacchariphagus*) eggs in field trials | ^51^ |

## **Temperate**

| Location | Ant Species | Crop | Ecosystem Service | Quantified Impact | Reference | |
| --- | --- | --- | --- | --- | --- | --- |
| USA (Kentucky) | Multiple ant species | Turfgrass | Pest control | Ants preyed on black cutworm and Japanese beetle larvae | ^52^ |  |
| USA (West Virginia) | Multiple ant species | Apple | Pest control | Ants significantly increased predation of codling moth (*Cydia pomonella*) eggs | ^53^ |  |
| Denmark | *Formica polyctena* | Apple | Pest control | *F. polyctena* reduced larvae of winter moth and apple scab incidence | ^54^ |  |

## **Mediterranean**

| Location | Ant Species | Crop | Ecosystem Service | Quantified Impact | Reference |
| --- | --- | --- | --- | --- | --- |
| Northeastern Spain | *Messor barbarus* | Cereal | Weed-seed removal | Ants removed 74-94% of seeds from small patches and 99-100% from large patches within 24 hours | ^55^ |
| Southwestern Spain | *Tapinoma nigerrimum* | Olive | Pest control | *T. nigerrimum* contributed strongly to potential pest control in olive groves | ^56^ |
| Western Australia | *Pheidole sp.* | Wheat-lupin rotation | Soil improvement | *Pheidole sp.* Increased soil water infiltration by up to sixfold under ponded conditions in farmland | ^57^ |
| Western Australia | Multiple ant species | Wheat | Soil improvement | Ants and termites increased wheat yield by 36% through improved water infiltration and soil nitrogen | ^58^ |
| Northeastern Spain | *M. barbarus* | Oats | Weed-seed removal | *M. barbarus* removed 78-100% of weed-seeds from cereal fields | ^59^ |
| Northeastern Spain | *M. barbarus* | Cereals | Weed-seed removal | *M. barbarus* removed an estimated 72% of weed seeds from cereal fields | ^60^ |

Notes

- Most quantified ecosystem service data come from tropical and subtropical perennial systems
- Very little published research exists for annual no-till crop systems in temperate region
- Ant species differ in the services they provide across agricultural systems
- This table summarizes field-based studies in agricultural systems. These studies quantified ant effects on crop yield, crop damage, and pest population density

References

1.	Peng R, and Christian K. The effect of the weaver ant, Oecophylla smaragdina (Hymenoptera: Formicidae), on the mango seed weevil, Sternochetus mangiferae (Coleoptera: Curculionidae), in mango orchards in the Northern Territory of Australia. International Journal of Pest Management. 2007;53:15–24. doi:10.1080/09670870600968859

2.	Peng R, and Christian K. The weaver ant, Oecophylla smaragdina (Hymenoptera: Formicidae), an effective biological control agent of the red-banded thrips, Selenothrips rubrocinctus (Thysanoptera: Thripidae) in mango crops in the Northern Territory of Australia. International Journal of Pest Management. 2004;50:107–14. doi:10.1080/09670870410001658125

3.	Anato FM, Wargui RB, Sinzogan AAC, Offenberg J, Adandonon A, Vayssières JF, et al. Reducing losses inflicted by insect pests on cashew, using weaver ants as a biological control agent. Agricultural and Forest Entomology. 2015;17:285–91. doi:10.1111/afe.12105

4.	Van Mele P. A historical review of research on the weaver ant Oecophylla in biological control. Agricultural and Forest Entomology. 2008;10:13–22. doi:10.1111/j.1461-9563.2007.00350.x

5.	Van Mele P, Vayssieres JF, Adandonon A, Sinzogan A. Ant cues affect the oviposition behaviour of fruit flies (Diptera: Tephritidae) in Africa. Physiological Entomology. 2009;34:256–61. doi:10.1111/j.1365-3032.2009.00685.x

6.	Agarwal VM, Rastogi N, Raju SVS. Impact of predatory ants on two lepidopteran insect pests in Indian cauliflower agroecosystems. Journal of Applied Entomology. 2007;131:493–500. doi:10.1111/j.1439-0418.2007.01197.x

7.	Jutsum AR, Cherrett JM, Fisher M. Interactions Between the Fauna of Citrus Trees in Trinidad and the Ants Atta cephalotes and Azteca Sp. Journal of Applied Ecology. 1981;18:187–95. doi:10.2307/2402488

8.	Bruneau De Miré P. An ant used in Cameroon in the fight against cocoa myrides: Wasmannia auropuncta Roger. Café, Cacao, Thé [Internet]. 1969 [cited 2025 Jun 17]. Located at: Cameroun. Available from: https://agritrop.cirad.fr/401803/

9.	Perfecto I, Castiñeiras A. Chapter 15 - Deployment of the predaceous ants and their conservation in agroecosystems. In: Barbosa P, editor. Conservation Biological Control [Internet]. San Diego: Academic Press; 1998 [cited 2025 May 1]. p. 269–89. Available from: https://www.sciencedirect.com/science/article/pii/B978012078147850061X doi:10.1016/B978-012078147-8/50061-X

10.	Yusa Y. PREDATION ON EGGS OF THE APPLE SNAIL POMACEA CANALICULATA (GASTROPODA: AMPULLARIIDAE) BY THE FIRE ANT SOLENOPSIS GEMINATA. J Molluscan Stud. 2001;67:275–9. doi:10.1093/mollus/67.3.275

11.	Wang L, Wang Z, Zeng L, Lu Y. Red imported fire ant invasion reduces the populations of two banana insect pests in South China. Sociobiology. 2016;63:889–93. doi:10.13102/sociobiology.v63i3.1035

12.	Bagny Beilhe L, Piou C, Tadu Z, Babin R. Identifying Ant-Mirid Spatial Interactions to Improve Biological Control in Cacao-Based Agroforestry System. Environ Entomol. 2018;47:551–8. doi:10.1093/ee/nvy018

13.	Abdulla NR, Rwegasira GM, Jensen KMV, Mwatawala MW, Offenberg J. Effect of supplementary feeding of Oecophylla longinoda on their abundance and predatory activities against cashew insect pests. Biocontrol Science and Technology. 2015;25:1333–45. doi:10.1080/09583157.2015.1057476

14.	Dwomoh EA, Afun JV, Ackonor JB, Agene V. Investigations on Oecophylla longinoda (Latreille) (Hymenoptera: Formicidae) as a biocontrol agent in the protection of cashew plantations. Pest Management Science. 2009;65:41–6. doi:10.1002/ps.1642

15.	Olotu MI, du Plessis H, Seguni ZS, Maniania NK. Efficacy of the African weaver ant Oecophylla longinoda (Hymenoptera: Formicidae) in the control of Helopeltis spp. (Hemiptera: Miridae) and Pseudotheraptus wayi (Hemiptera: Coreidae) in cashew crop in Tanzania. Pest Management Science. 2013;69:911–8. doi:10.1002/ps.3451

16.	Peng RK, Christian K, Gibb K. The effect of the green ant, Oecophylla smaragdina (Hymenoptera: Formicidae), on insect pests of cashew trees in Australia. Bulletin of Entomological Research. 1995;85:279–84. doi:10.1017/S0007485300034374

17.	Ativor IN, Afreh-Nuamah K, Billah M, Obeng-Ofori D. Weaver Ant, Oecophylla longinoda (Latreille) (Hymenoptera: Formicidae) activity reduces fruit fly damage in citrus orchards. Journal of Agricultural Science and Technology. 2012;A 2:449–58.

18.	Sporleder M, Rapp G. The effect of Oecophylla longinoda (Latr.) (Hym., Formicidae) on coconut palm productivity with respect to Pseudotheraptus wayi Brown (Hem., Coreidae) damage in Zanzibar. Journal of Applied Entomology. 1998;122:475–81. doi:10.1111/j.1439-0418.1998.tb01530.x

19.	Jiménez-Soto E, Cruz-Rodríguez JA, Vandermeer J, Perfecto I. Hypothenemus hampei (Coleoptera: Curculionidae) and its interactions with Azteca instabilis and Pheidole synanthropica (Hymenoptera: Formicidae) in a shade coffee agroecosystem. Environ Entomol. 2013;42:915–24. doi:10.1603/EN12202 PubMed PMID: 24331603.

20.	Pardee GL, Philpott SM. Cascading Indirect Effects in a Coffee Agroecosystem: Effects of Parasitic Phorid Flies on Ants and the Coffee Berry Borer in a High-Shade and Low-Shade Habitat. Environ Entomol. 2011;40:581–8. doi:10.1603/EN11015

21.	Morris JR, Vandermeer J, Perfecto I. A Keystone Ant Species Provides Robust Biological Control of the Coffee Berry Borer Under Varying Pest Densities. Doucet D, editor. PLoS ONE. 2015;10:e0142850. doi:10.1371/journal.pone.0142850

22.	Morris JR, Perfecto I. An aggressive nonconsumptive effect mediates pest control and multipredator interactions in a coffee agroecosystem. Ecological Applications. 2022;32:e2653. doi:10.1002/eap.2653

23.	Eubanks MD. Estimates of the Direct and Indirect Effects of Red Imported Fire Ants on Biological Control in Field Crops. Biological Control. 2001;21:35–43. doi:10.1006/bcon.2001.0923

24.	Agarwal VM, Rastogi N. Deterrent effect of a guild of extrafloral nectary-visiting ant species on Raphidopalpa foveicollis, a major insect pest of sponge gourd, Luffa cylindrica. Entomologia Experimentalis et Applicata. 2008;128:303–11. doi:10.1111/j.1570-7458.2008.00727.x

25.	Sekamatte BM, Ogenga-Latigo M, Russell-Smith A. Effects of maize–legume intercrops on termite damage to maize, activity of predatory ants and maize yields in Uganda. Crop Protection. 2003;22:87–93. doi:10.1016/S0261-2194(02)00115-1

26.	Knutson AE, Campos M. Effect of Red Imported Fire Ant, Solenopsis invicta1, on Abundance of Corn Earworm, Helicoverpa zea2, on Maize in Texas. swen. 2008;33:1–13. doi:10.3958/0147-1724-33.1.1

27.	Abdulla NR, Rwegasira GM, Mwatawala MW. Efficacy of African weaver ant, Oecophylla longinoda (Hymenoptera: Formicidae) in reducing losses due to frugivorous fruit flies (Diptera: Tephritidae) in smallholder mango production systems in Eastern Tanzania. Biocontrol Science and Technology. 2017;27:1205–19. doi:10.1080/09583157.2017.1391173

28.	Abdulla NR, Rwegasira GM, Jensen KMV, Mwatawala MW, Offenberg J. Control of mango seed weevils (Sternochetus mangiferae) using the African Weaver Ant (Oecophylla longinoda Latreille) (Hymenoptera: Formicidae). Journal of Applied Entomology. 2016;140:500–6. doi:10.1111/jen.12260

29.	Adandonon A, Vayssières J, Sinzogan A, Van Mele P. Density of pheromone sources of the weaver ant Oecophylla longinoda (Hymenoptera Formicidae) affects oviposition behaviour and damage by mango fruit flies (Diptera Tephritidae). Int J Pest Manag. 2009;55:285–92.

30.	Anikwe JC, Omoloye AA, Okelana FA. Studies of the Ant-plant Mutualism in the Nigerian Cocoa Agroecology. 2007.

31.	Perfecto I. Indirect and Direct Effects in a Tropical Agroecosystem: The Maize‐Pest‐Ant System in Nicaragua. Ecology. 1990;71:2125–34. doi:10.2307/1938626

32.	Styrsky JD, Eubanks MD. A facultative mutualism between aphids and an invasive ant increases plant reproduction. Ecological Entomology. 2010;35:190–9. doi:10.1111/j.1365-2311.2009.01172.x

33.	Bisseleua DHB, Begoude D, Tonnang H, Vidal S. Ant-mediated ecosystem services and disservices on marketable yield in cocoa agroforestry systems. Agriculture, Ecosystems & Environment. 2017;247:409–17. doi:10.1016/j.agee.2017.07.004

34.	Elvin MK, Stimac JL, Whitcomb WH. Estimating Rates of Arthropod Predation on Velvetbean Caterpillar Larvae in Soybeans. The Florida Entomologist. 1983;66:319–30. doi:10.2307/3494127

35.	Fernandes WD, Oliveira PS, Carvalho SL, Habib MEM. Pheidole ants as potential biological control agents of the boll weevil, Anthonomus grandis (Col., Curculionidae), in Southeast Brazil. Journal of Applied Entomology. 1994;118:437–41. doi:10.1111/j.1439-0418.1994.tb00822.x

36.	Sterling WL. Fortuitous Biological Suppression of the Boll Weevil by the Red Imported Fire Ant. Environ Entomol. 1978;7:564–8. doi:10.1093/ee/7.4.564

37.	Van Den Berg H, Bagus A, Hassan K, Muhammad A, Zega S. Predation and parasitism on eggs of two pod‐sucking bugs, Nezara viridula and Piezodorus hybneri in soybean. International Journal of Pest Management. 1995;41:134–42. doi:10.1080/09670879509371938

38.	Way MJ, Javier G, Heong KL. The role of ants, especially the fire ant, Solenopsis geminata (Hymenoptera: Formicidae), in the biological control of tropical upland rice pests. Bulletin of Entomological Research. 2002;92:431–7. doi:10.1079/BER2002185

39.	Armbrecht I, Gallego MC. Testing ant predation on the coffee berry borer in shaded and sun coffee plantations in Colombia. Entomologia Experimentalis et Applicata. 2007;124:261–7. doi:10.1111/j.1570-7458.2007.00574.x

40.	Oliveira RDFD, Almeida LCD, Souza DRD, Munhae CB, Bueno OC, Morini MSDC. Ant diversity (Hymenoptera: Formicidae) and predation by ants on the different stages of the sugarcane borer life cycle Diatraea saccharalis (Lepidoptera: Crambidae). EJE. 2013;109:381–7.

41.	Mollot G, Tixier P, Lescourret F, Quilici S, Duyck PF. New primary resource increases predation on a pest in a banana agroecosystem. Agricultural and Forest Entomology. 2012;14:317–23. doi:10.1111/j.1461-9563.2012.00571.x

42.	Pierre E, Idris A. Studies on the predatory activities of Oecophylla smaragdina (Hymenoptera: Formicidae) on Pteroma pendula (Lepidoptera: Psychidae) in oil palm plantations in Teluk Intan, Perak (Malaysia). Asian Myrmecology. 2013;5:163–76.

43.	Daha L, Amin N, Abdullah T. The Study on the Roles of Predators on Asian Corn Stem Borer, *Ostrinia furnacalis* Guenee (Lepidoptera: Pyralidae). OnLine Journal of Biological Sciences. 2016;16:49–55. doi:10.3844/ojbsci.2016.49.55

44.	Gossler O, Lange D, Wedson D. Can the absence of ants interfere in the arthropods abundance on corn plants (Zea mays L.-Poaceae)? Plant Production and Crop Protection. Comunicata Scientiae. 2017;8:9–16. doi:10.14295/CS.v8i1.1730

45.	Koffi D, Kyerematen R, Eziah VY, Agboka K, Adom M, Goergen G, et al. Natural Enemies of the Fall Armyworm, Spodoptera frugiperda (J.E. Smith) (Lepidoptera: Noctuidae) in Ghana. flen. 2020;103:85–90. doi:10.1653/024.103.0414

46.	Ahissou BR, W Mathieu S, Bonzi S, Baimey H, Somda I, Bokonon-Ganta A, et al. Natural enemies of the fall armyworm Spodoptera frugiperda (Smith) (Lepidoptera: Noctuidae) in Burkina Faso. Tropicultura. 2021. doi:10.25518/2295-8010.1881

47.	Seagraves M, McPherson R. Monitoring Red Imported Fire Ant (Hymenoptera: Formicidae) Foraging Behavior and Impact on Foliage-Dwelling Arthropods on Soybean Produced Under Three Ant Suppression Regimes. Journal of Entomological Science. 2006;41:374–84. doi:10.18474/0749-8004-41.4.374

48.	Löhr B, Oswald S. An integrated pest management approach towards the control of the low-density coconut pest, Pseudotheraptus wayi Brown (Hemiptera: Coreidae). 1990.

49.	Peng RK, Christian K. The control efficacy of the weaver ant, Oecophylla smaragdina (Hymenoptera: Formicidae), on the mango leafhopper, Idioscopus nitidulus (Hemiptera: Cicadellidea) in mango orchards in the Northern Territory. International Journal of Pest Management. 2005;51:297–304. doi:10.1080/09670870500151689

50.	Diamé L, Grechi I, Rey JY, Sané C a. B, Diatta P, Vayssières JF, et al. Influence of Oecophylla longinoda Latreille, 1802 (Hymenoptera: Formicidae) on Mango Infestation by Bactrocera dorsalis (Hendel) (Diptera: Tephritidae) in Relation to Senegalese Orchard Design and Management Practices. afen. 2015;23:294–305. doi:10.4001/003.023.0207

51.	Goebel FR, Fernandez E, Begue JM, Alauzet C. Predation by Pheidole megacephala (Fabricius) (Hym. : Formicidae) on eggs of the sugarcane stem borer Chilo sacchariphagus (Bojer) (Lep. : Pyralidae) in Reunion Island. Annales- Societe Entomologique de France. 1999;35:440–2.

52.	López R, Potter DA. Ant Predation on Eggs and Larvae of the Black Cutworm (Lepidoptera: Noctuidae) and Japanese Beetle (Coleoptera: Scarabaeidae) in Turfgrass. Environmental Entomology. 2000;29:116–25. doi:10.1603/0046-225X-29.1.116

53.	Mathews CR, Bottrell DG, Brown MW. Habitat manipulation of the apple orchard floor to increase ground-dwelling predators and predation of *Cydia pomonella* (L.) (Lepidoptera: Tortricidae). Biological Control. 2004;30:265–73. doi:10.1016/j.biocontrol.2003.11.006

54.	Offenberg J, Nielsen J, Damgaard C. Wood Ant (Formica polyctena) Services and Disservices in a Danish Apple Plantation. Sociobiology. 2019;66:247. doi:10.13102/sociobiology.v66i2.4251

55.	Westerman P, Atanackovic V, Torra J. Effect of weed patch size on seed removal by harvester ants. Tagungsband : 26 Deutsche Arbeitsbesprechung über Fragen der Unkrautbiologie und - bekämpfung ; 11-13 März 2014, Braunschweig. 2014;451–5. doi:10.5073/jka.2014.443.056

56.	Martínez-Núñez C, Rey PJ, Salido T, Manzaneda AJ, Camacho FM, Isla J. Ant community potential for pest control in olive groves: Management and landscape effects. Agriculture, Ecosystems & Environment. 2021;305:107185. doi:10.1016/j.agee.2020.107185

57.	De Bruyn LAL, Conacher AJ. The effect of ant biopores on water infiltration in soils in undisturbed bushland and in farmland in a semi-arid environment. Pedobiologia. 1994;38:193–207. doi:10.1016/S0031-4056(24)00122-7

58.	Evans TA, Dawes TZ, Ward PR, Lo N. Ants and termites increase crop yield in a dry climate. Nat Commun. 2011;2:262. doi:10.1038/ncomms1257

59.	Torra J, Atanackovic V, Blanco-Moreno JM, Royo-Esnal A, Westerman PR. Effect of patch size on seed removal by harvester ants. Weed Research. 2016;56:14–21. doi:10.1111/wre.12179

60.	Westerman PR, Atanackovic V, Royo-Esnal A, Torra J. Differential weed seed removal in dryland cereals. Arthropod-Plant Interactions. 2012;6:591–9. doi:10.1007/s11829-012-9211-6
